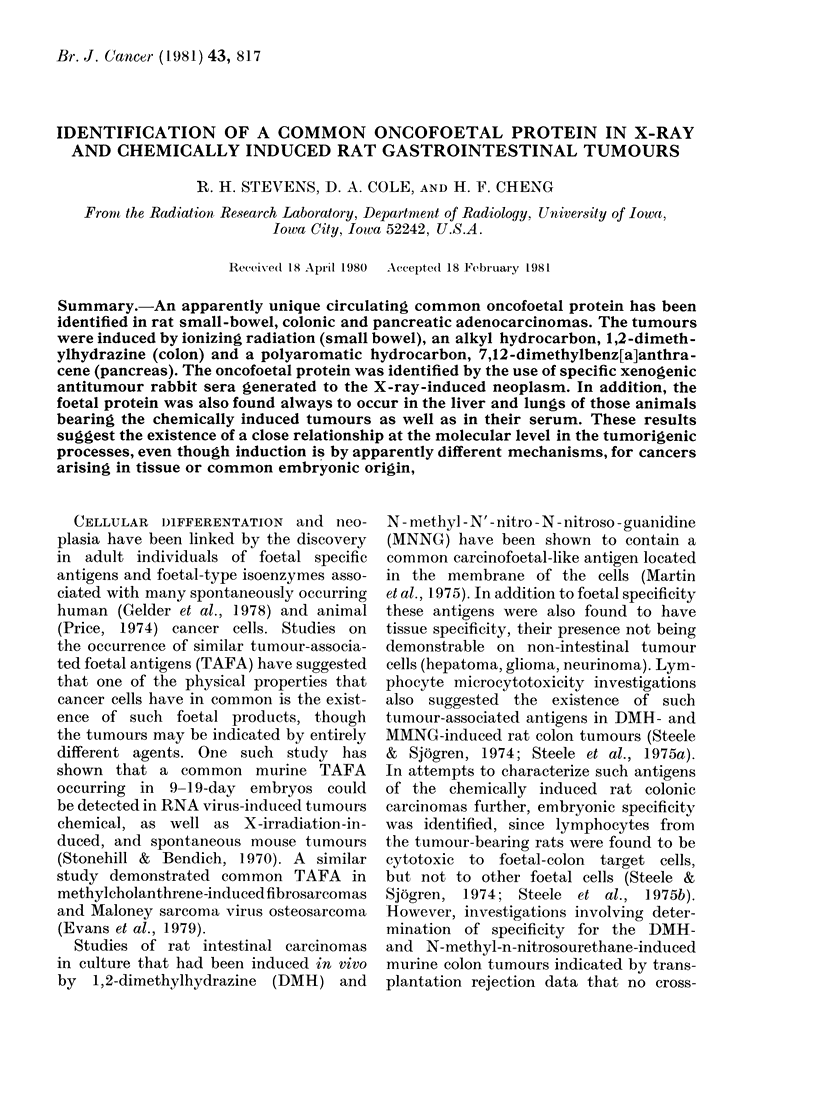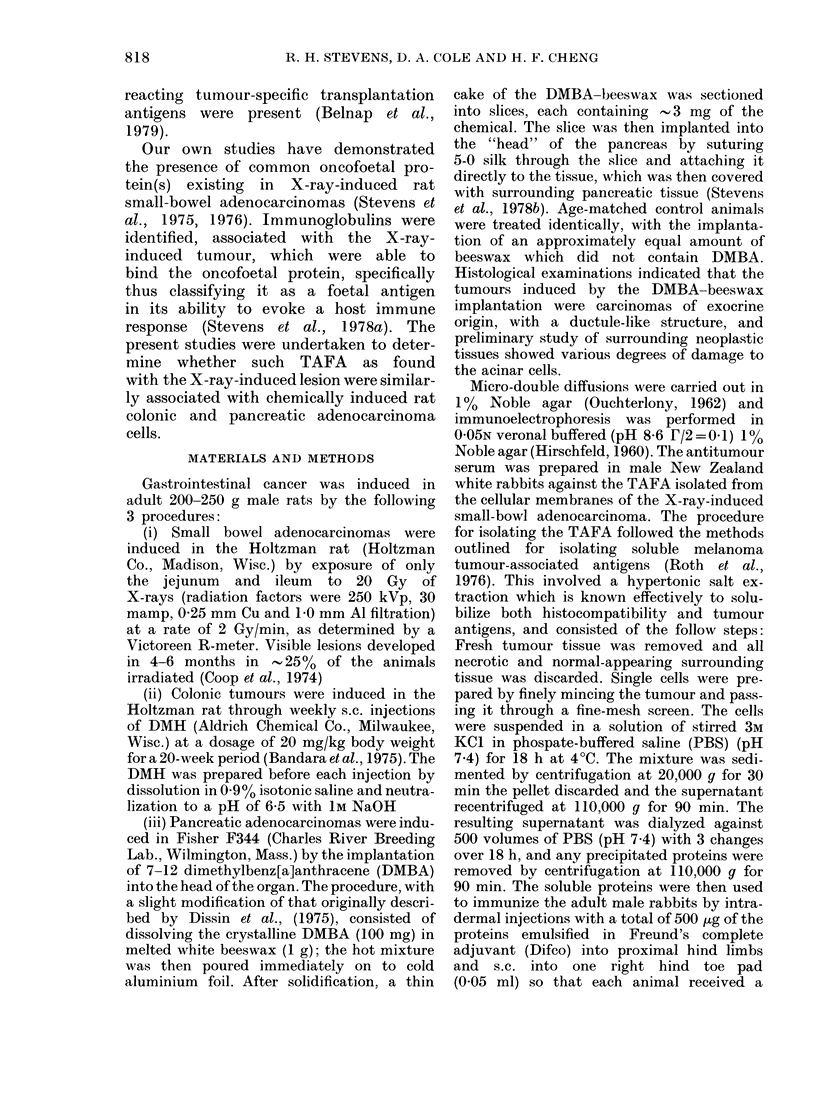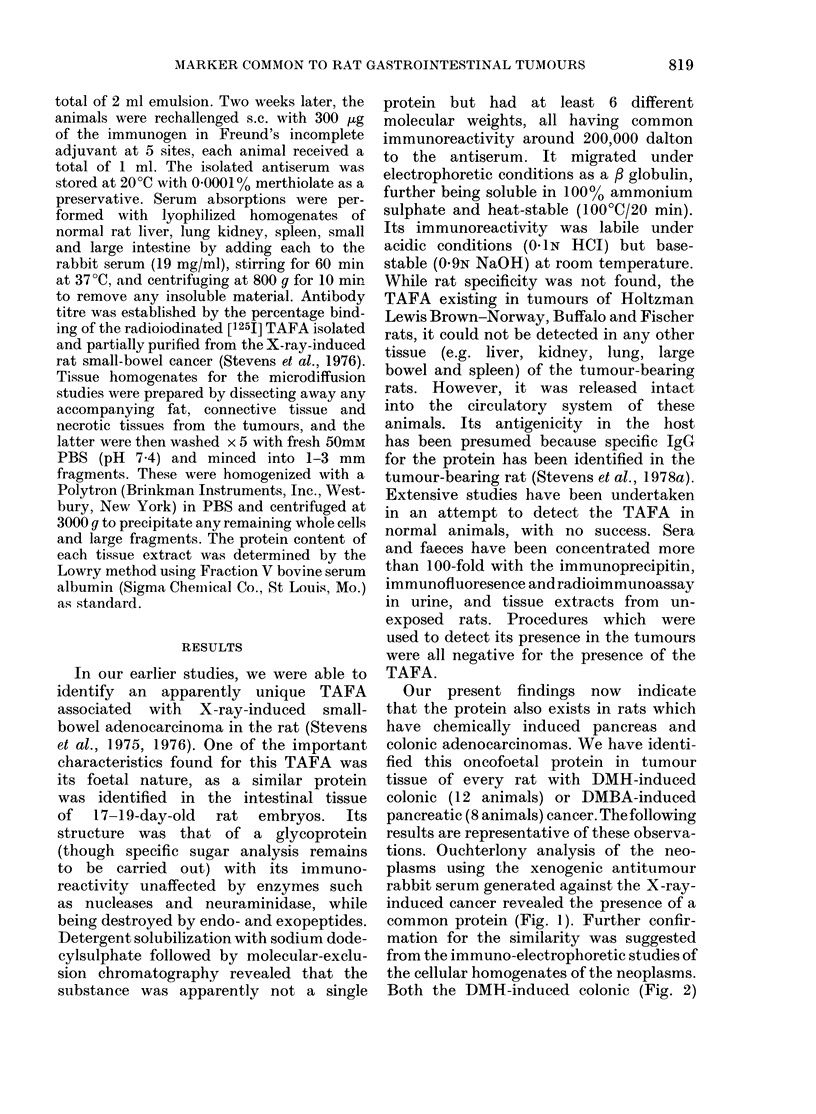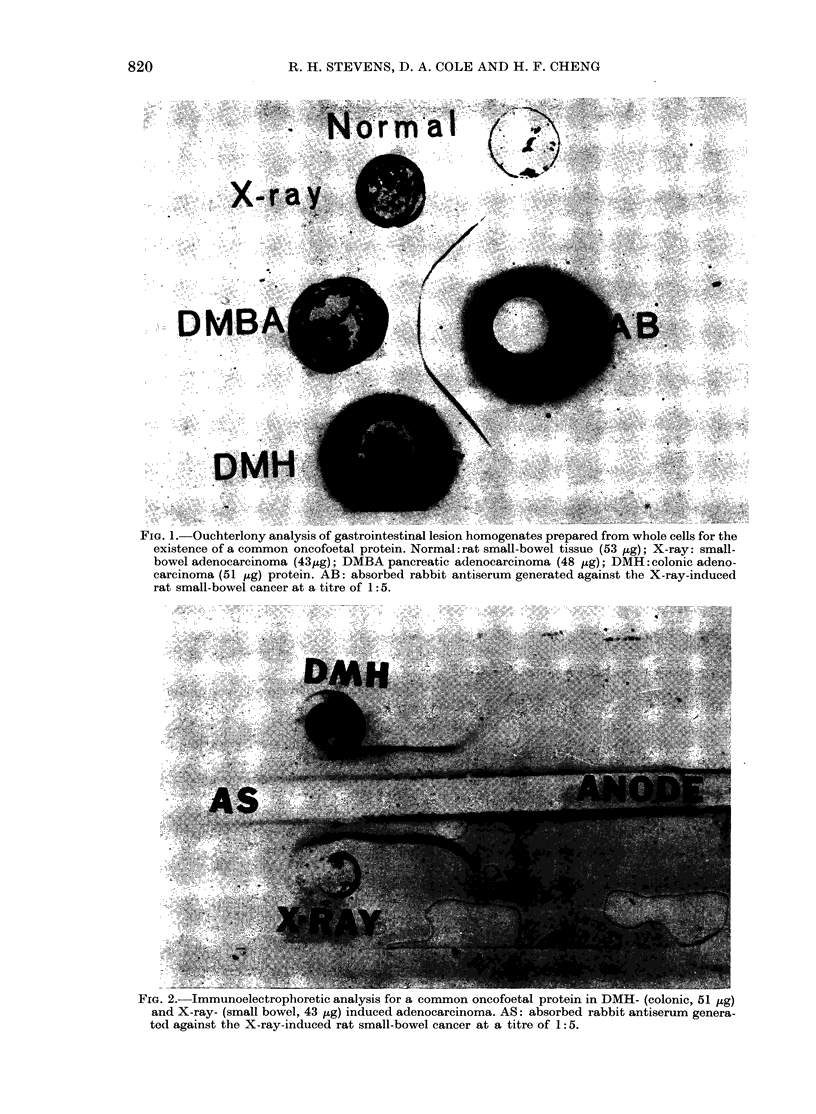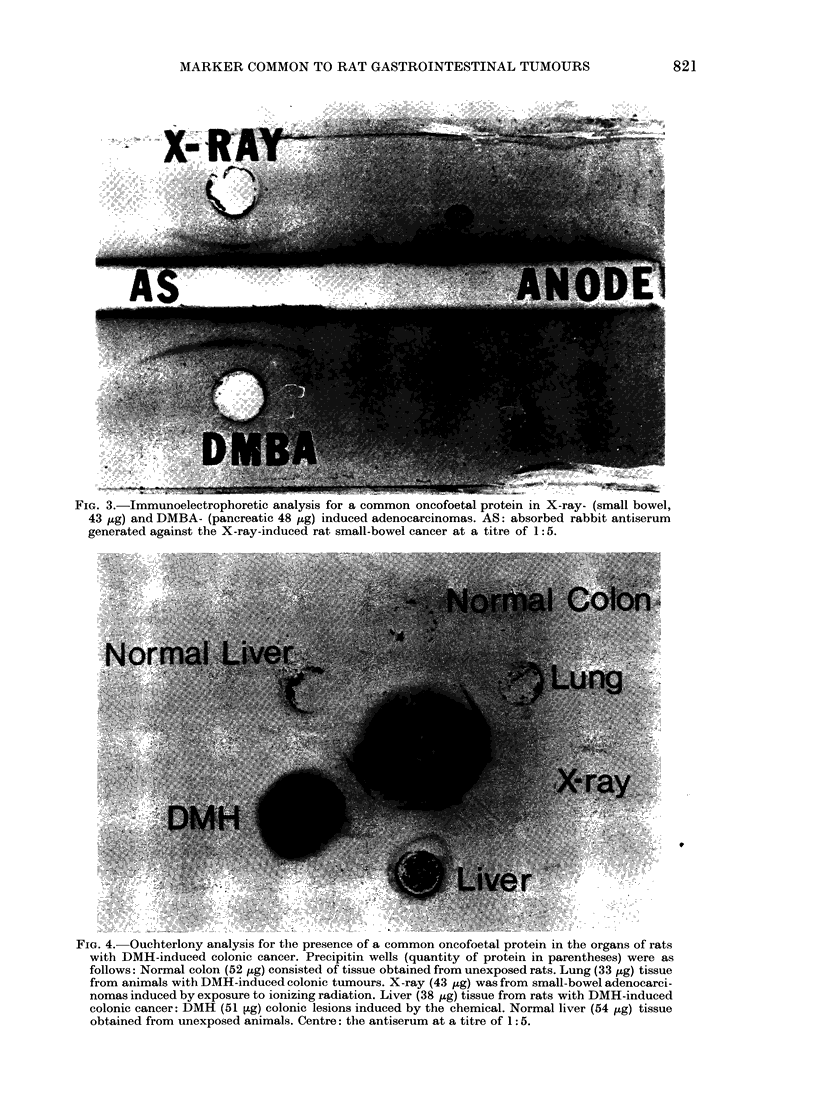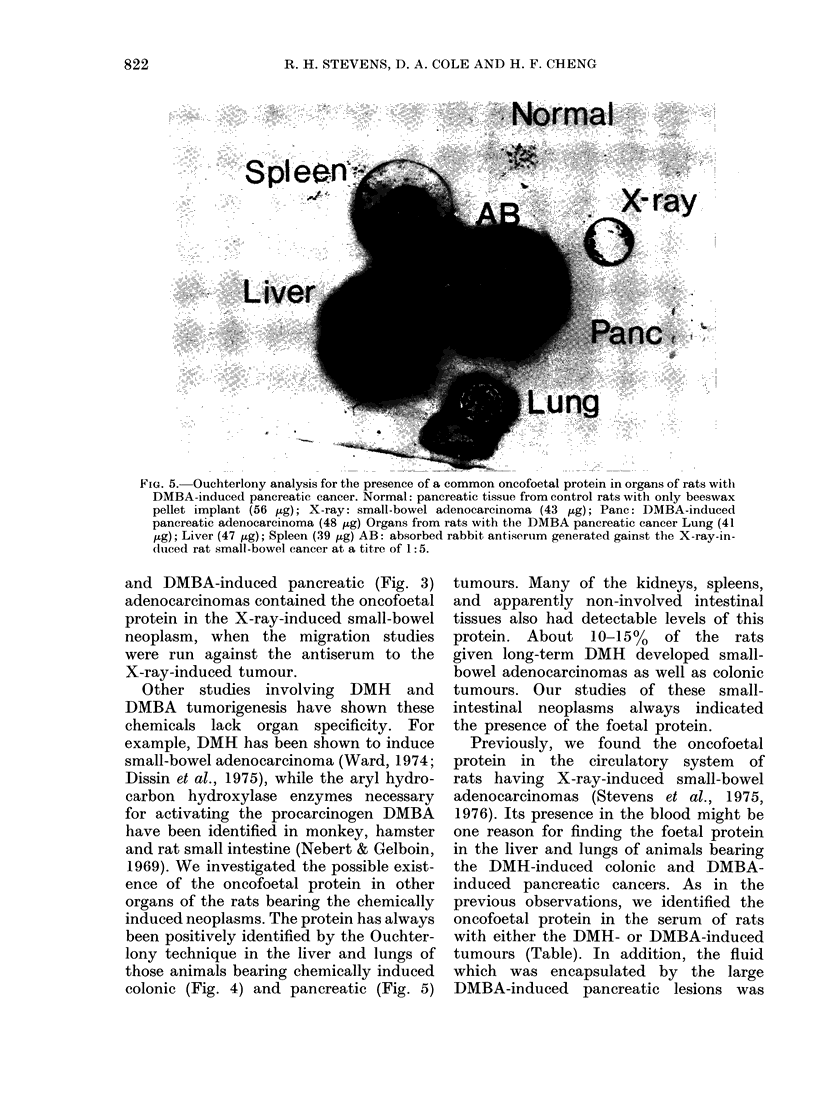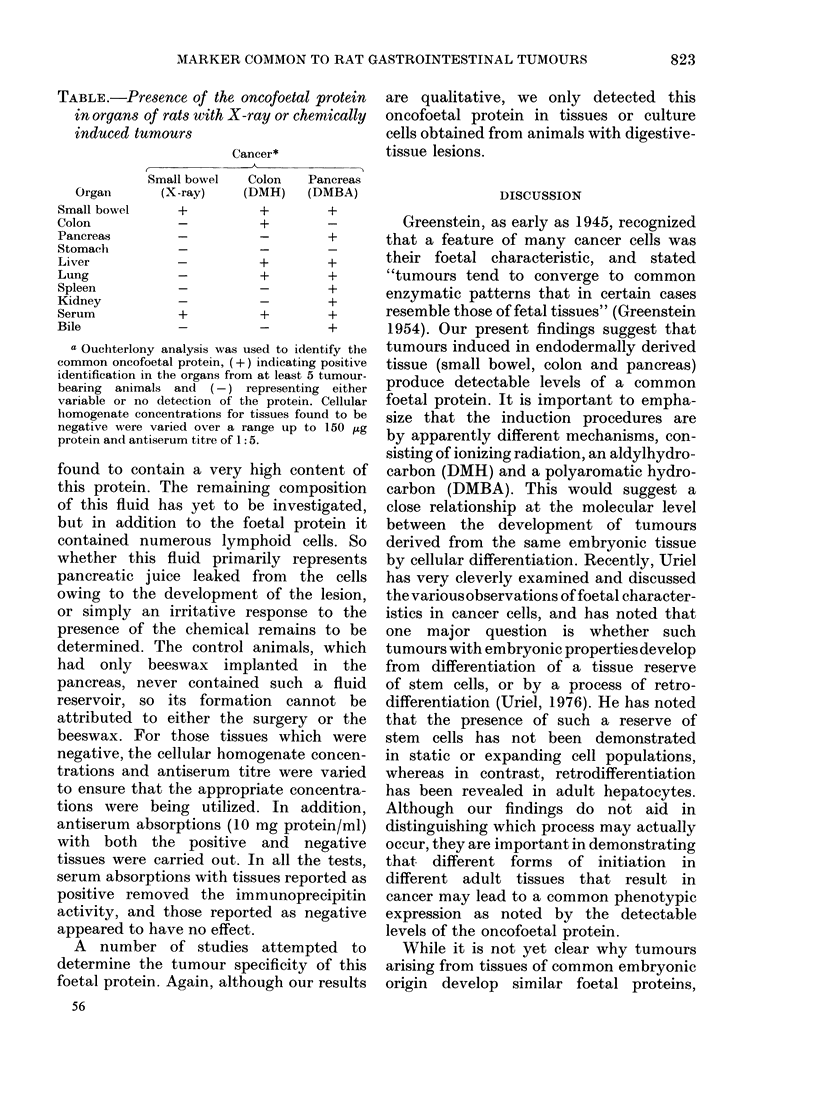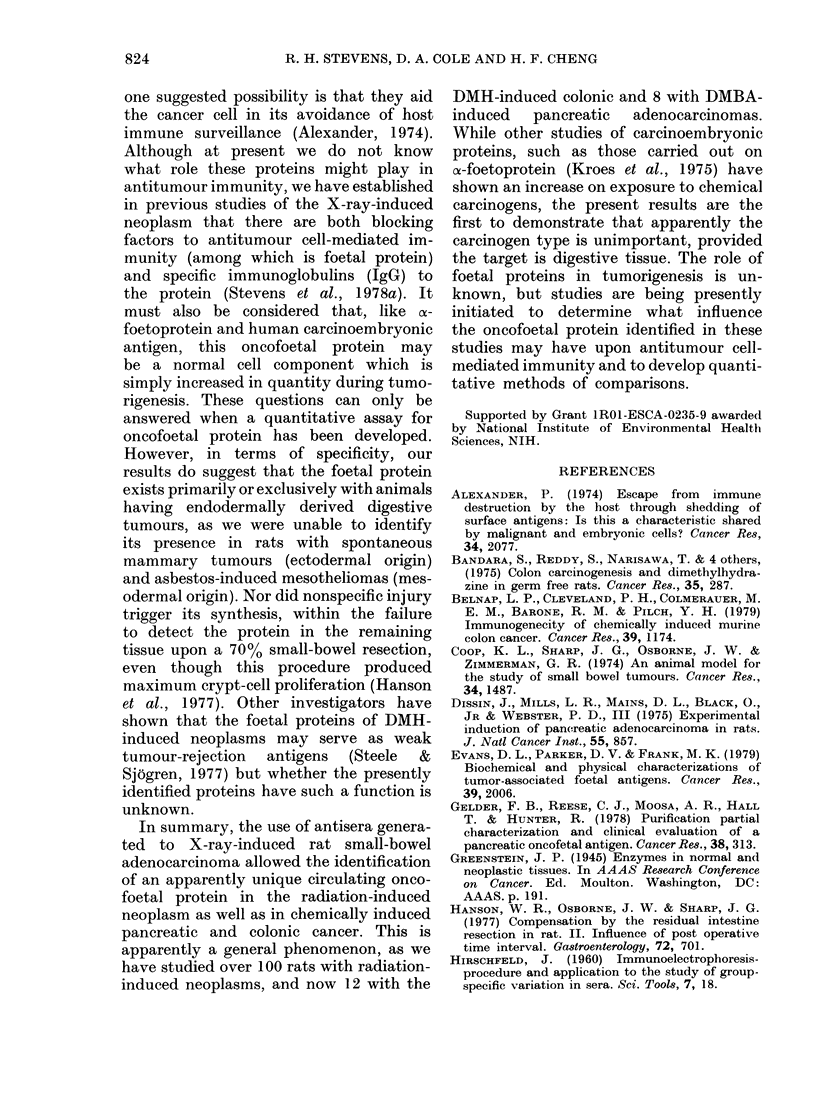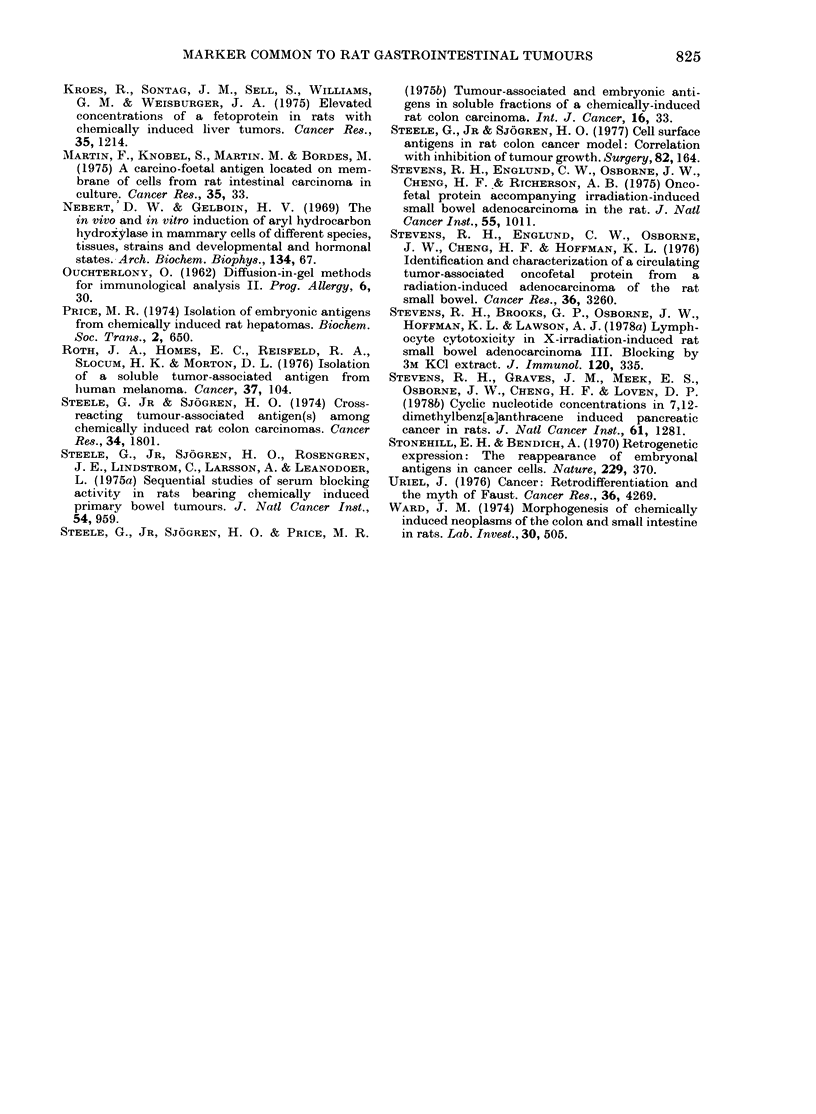# Identification of a common oncofoetal protein in x-ray and chemically induced rat gastrointestinal tumours.

**DOI:** 10.1038/bjc.1981.120

**Published:** 1981-06

**Authors:** R. H. Stevens, D. A. Cole, H. F. Cheng

## Abstract

**Images:**


Br. J. Cancer (1981) 43, 817

### IDENTIFICATION OF A COMMON ONCOFOETAL PROTEIN IN X-RAY AND CHEMICALLY INDUCED RAT GASTROINTESTINAL TUMOURS

R. H. STEVENS, D. A. COLE, AND H. F. CHENG

*From the Radiation Research Laboratory, Department of Radiology, University of Iowa, Iowa City, Iowa 52242, U.S.A.*

Received 18 April 1980 Accepted 18 February 1981

**Summary.—An apparently unique circulating common oncofoetal protein has been identified in rat small-bowel, colonic and pancreatic adenocarcinomas. The tumours were induced by ionizing radiation (small bowel), an alkyl hydrocarbon, 1,2-dimethylhydrazine (colon) and a polyaromatic hydrocarbon, 7,12-dimethylbenz[a]anthracene (pancreas). The oncofoetal protein was identified by the use of specific xenogenic antitumour rabbit sera generated to the X-ray-induced neoplasm. In addition, the foetal protein was also found always to occur in the liver and lungs of those animals bearing the chemically induced tumours as well as in their serum. These results suggest the existence of a close relationship at the molecular level in the tumorigenic processes, even though induction is by apparently different mechanisms, for cancers arising in tissue or common embryonic origin,**

Cellular differentation and neoplasia have been linked by the discovery in adult individuals of foetal specific antigens and foetal-type isoenzymes associated with many spontaneously occurring human (Gelder *et al.*, 1978) and animal (Price, 1974) cancer cells. Studies on the occurrence of similar tumour-associated foetal antigens (TAFA) have suggested that one of the physical properties that cancer cells have in common is the existence of such foetal products, though the tumours may be indicated by entirely different agents. One such study has shown that a common murine TAFA occurring in 9–19-day embryos could be detected in RNA virus-induced tumours chemical, as well as X-irradiation-induced, and spontaneous mouse tumours (Stonehill & Bendich, 1970). A similar study demonstrated common TAFA in methylcholanthrene-induced fibrosarcomas and Maloney sarcoma virus osteosarcoma (Evans *et al.*, 1979).

Studies of rat intestinal carcinomas in culture that had been induced *in vivo* by 1,2-dimethylhydrazine (DMH) and N-methyl-N′-nitro-N-nitroso-guanidine (MNNG) have been shown to contain a common carcinofoetal-like antigen located in the membrane of the cells (Martin *et al.*, 1975). In addition to foetal specificity these antigens were also found to have tissue specificity, their presence not being demonstrable on non-intestinal tumour cells (hepatoma, glioma, neurinoma). Lymphocyte microcytotoxicity investigations also suggested the existence of such tumour-associated antigens in DMH- and MMNG-induced rat colon tumours (Steele & Sjögren, 1974; Steele *et al.*, 1975*a*). In attempts to characterize such antigens of the chemically induced rat colonic carcinomas further, embryonic specificity was identified, since lymphocytes from the tumour-bearing rats were found to be cytotoxic to foetal-colon target cells, but not to other foetal cells (Steele & Sjögren, 1974; Steele *et al.*, 1975*b*). However, investigations involving determination of specificity for the DMH- and N-methyl-n-nitrosourethane-induced murine colon tumours indicated by transplantation rejection data that no crossreacting tumour-specific transplantation antigens were present (Belnap *et al.*, 1979).

Our own studies have demonstrated the presence of common oncofoetal protein(s) existing in X-ray-induced rat small-bowel adenocarcinomas (Stevens *et al.*, 1975, 1976). Immunoglobulins were identified, associated with the X-ray-induced tumour, which were able to bind the oncofoetal protein, specifically thus classifying it as a foetal antigen in its ability to evoke a host immune response (Stevens *et al.*, 1978*a*). The present studies were undertaken to determine whether such TAFA as found with the X-ray-induced lesion were similarly associated with chemically induced rat colonic and pancreatic adenocarcinoma cells.

### MATERIALS AND METHODS

Gastrointestinal cancer was induced in adult 200–250 g male rats by the following 3 procedures:

(i) Small bowel adenocarcinomas were induced in the Holtzman rat (Holtzman Co., Madison, Wisc.) by exposure of only the jejunum and ileum to 20 Gy of X-rays (radiation factors were 250 kVp, 30 mamp, 0·25 mm Cu and 1·0 mm Al filtration) at a rate of 2 Gy/min, as determined by a Victoreen R-meter. Visible lesions developed in 4–6 months in ~25% of the animals irradiated (Coop *et al.*, 1974)

(ii) Colonic tumours were induced in the Holtzman rat through weekly s.c. injections of DMH (Aldrich Chemical Co., Milwaukee, Wisc.) at a dosage of 20 mg/kg body weight for a 20-week period (Bandara *et al.*, 1975). The DMH was prepared before each injection by dissolution in 0·9% isotonic saline and neutralization to a pH of 6·5 with 1M NaOH

(iii) Pancreatic adenocarcinomas were induced in Fisher F344 (Charles River Breeding Lab., Wilmington, Mass.) by the implantation of 7–12 dimethylbenz[a]anthracene (DMBA) into the head of the organ. The procedure, with a slight modification of that originally described by Dissin *et al.*, (1975), consisted of dissolving the crystalline DMBA (100 mg) in melted white beeswax (1 g); the hot mixture was then poured immediately on to cold aluminium foil. After solidification, a thin cake of the DMBA–beeswax was sectioned into slices, each containing ~3 mg of the chemical. The slice was then implanted into the "head" of the pancreas by suturing 5-0 silk through the slice and attaching it directly to the tissue, which was then covered with surrounding pancreatic tissue (Stevens *et al.*, 1978*b*). Age-matched control animals were treated identically, with the implantation of an approximately equal amount of beeswax which did not contain DMBA. Histological examinations indicated that the tumours induced by the DMBA–beeswax implantation were carcinomas of exocrine origin, with a ductule-like structure, and preliminary study of surrounding neoplastic tissues showed various degrees of damage to the acinar cells.

Micro-double diffusions were carried out in 1% Noble agar (Ouchterlony, 1962) and immunoelectrophoresis was performed in 0·05N veronal buffered (pH 8·6 $\Gamma/2=0{\cdot}1$) 1% Noble agar (Hirschfeld, 1960). The antitumour serum was prepared in male New Zealand white rabbits against the TAFA isolated from the cellular membranes of the X-ray-induced small-bowl adenocarcinoma. The procedure for isolating the TAFA followed the methods outlined for isolating soluble melanoma tumour-associated antigens (Roth *et al.*, 1976). This involved a hypertonic salt extraction which is known effectively to solubilize both histocompatibility and tumour antigens, and consisted of the follow steps: Fresh tumour tissue was removed and all necrotic and normal-appearing surrounding tissue was discarded. Single cells were prepared by finely mincing the tumour and passing it through a fine-mesh screen. The cells were suspended in a solution of stirred 3M KCl in phospate-buffered saline (PBS) (pH 7·4) for 18 h at 4°C. The mixture was sedimented by centrifugation at 20,000 *g* for 30 min the pellet discarded and the supernatant recentrifuged at 110,000 *g* for 90 min. The resulting supernatant was dialyzed against 500 volumes of PBS (pH 7·4) with 3 changes over 18 h, and any precipitated proteins were removed by centrifugation at 110,000 *g* for 90 min. The soluble proteins were then used to immunize the adult male rabbits by intradermal injections with a total of 500 μg of the proteins emulsified in Freund's complete adjuvant (Difco) into proximal hind limbs and s.c. into one right hind toe pad (0·05 ml) so that each animal received a total of 2 ml emulsion. Two weeks later, the animals were rechallenged s.c. with 300 $\mu g$ of the immunogen in Freund's incomplete adjuvant at 5 sites, each animal received a total of 1 ml. The isolated antiserum was stored at 20°C with 0·0001% merthiolate as a preservative. Serum absorptions were performed with lyophilized homogenates of normal rat liver, lung kidney, spleen, small and large intestine by adding each to the rabbit serum (19 mg/ml), stirring for 60 min at 37°C, and centrifuging at 800 $g$ for 10 min to remove any insoluble material. Antibody titre was established by the percentage binding of the radioiodinated [$^{125}$I] TAFA isolated and partially purified from the X-ray-induced rat small-bowel cancer (Stevens *et al.*, 1976). Tissue homogenates for the microdiffusion studies were prepared by dissecting away any accompanying fat, connective tissue and necrotic tissues from the tumours, and the latter were then washed ×5 with fresh 50mM PBS (pH 7·4) and minced into 1–3 mm fragments. These were homogenized with a Polytron (Brinkman Instruments, Inc., Westbury, New York) in PBS and centrifuged at 3000 $g$ to precipitate any remaining whole cells and large fragments. The protein content of each tissue extract was determined by the Lowry method using Fraction V bovine serum albumin (Sigma Chemical Co., St Louis, Mo.) as standard.

### RESULTS

In our earlier studies, we were able to identify an apparently unique TAFA associated with X-ray-induced small-bowel adenocarcinoma in the rat (Stevens *et al.*, 1975, 1976). One of the important characteristics found for this TAFA was its foetal nature, as a similar protein was identified in the intestinal tissue of 17–19-day-old rat embryos. Its structure was that of a glycoprotein (though specific sugar analysis remains to be carried out) with its immunoreactivity unaffected by enzymes such as nucleases and neuraminidase, while being destroyed by endo- and exopeptides. Detergent solubilization with sodium dodecylsulphate followed by molecular-exclusion chromatography revealed that the substance was apparently not a single protein but had at least 6 different molecular weights, all having common immunoreactivity around 200,000 dalton to the antiserum. It migrated under electrophoretic conditions as a $\beta$ globulin, further being soluble in 100% ammonium sulphate and heat-stable (100°C/20 min). Its immunoreactivity was labile under acidic conditions (0·1N HCl) but base-stable (0·9N NaOH) at room temperature. While rat specificity was not found, the TAFA existing in tumours of Holtzman Lewis Brown–Norway, Buffalo and Fischer rats, it could not be detected in any other tissue (e.g. liver, kidney, lung, large bowel and spleen) of the tumour-bearing rats. However, it was released intact into the circulatory system of these animals. Its antigenicity in the host has been presumed because specific IgG for the protein has been identified in the tumour-bearing rat (Stevens *et al.*, 1978*a*). Extensive studies have been undertaken in an attempt to detect the TAFA in normal animals, with no success. Sera and faeces have been concentrated more than 100-fold with the immunoprecipitin, immunofluoresence and radioimmunoassay in urine, and tissue extracts from unexposed rats. Procedures which were used to detect its presence in the tumours were all negative for the presence of the TAFA.

Our present findings now indicate that the protein also exists in rats which have chemically induced pancreas and colonic adenocarcinomas. We have identified this oncofoetal protein in tumour tissue of every rat with DMH-induced colonic (12 animals) or DMBA-induced pancreatic (8 animals) cancer. The following results are representative of these observations. Ouchterlony analysis of the neoplasms using the xenogenic antitumour rabbit serum generated against the X-ray-induced cancer revealed the presence of a common protein (Fig. 1). Further confirmation for the similarity was suggested from the immuno-electrophoretic studies of the cellular homogenates of the neoplasms. Both the DMH-induced colonic (Fig. 2)

FIG. 1.—Ouchterlony analysis of gastrointestinal lesion homogenates prepared from whole cells for the existence of a common oncofoetal protein. Normal: rat small-bowel tissue (53 μg); X-ray: small-bowel adenocarcinoma (43μg); DMBA pancreatic adenocarcinoma (48 μg); DMH: colonic adenocarcinoma (51 μg) protein. AB: absorbed rabbit antiserum generated against the X-ray-induced rat small-bowel cancer at a titre of 1:5.

FIG. 2.—Immunoelectrophoretic analysis for a common oncofoetal protein in DMH- (colonic, 51 μg) and X-ray- (small bowel, 43 μg) induced adenocarcinoma. AS: absorbed rabbit antiserum generated against the X-ray-induced rat small-bowel cancer at a titre of 1:5.

FIG. 3.—Immunoelectrophoretic analysis for a common oncofoetal protein in X-ray- (small bowel, 43 μg) and DMBA- (pancreatic 48 μg) induced adenocarcinomas. AS: absorbed rabbit antiserum generated against the X-ray-induced rat small-bowel cancer at a titre of 1:5.

FIG. 4.—Ouchterlony analysis for the presence of a common oncofoetal protein in the organs of rats with DMH-induced colonic cancer. Precipitin wells (quantity of protein in parentheses) were as follows: Normal colon (52 μg) consisted of tissue obtained from unexposed rats. Lung (33 μg) tissue from animals with DMH-induced colonic tumours. X-ray (43 μg) was from small-bowel adenocarcinomas induced by exposure to ionizing radiation. Liver (38 μg) tissue from rats with DMH-induced colonic cancer: DMH (51 μg) colonic lesions induced by the chemical. Normal liver (54 μg) tissue obtained from unexposed animals. Centre: the antiserum at a titre of 1:5.

FIG. 5.—Ouchterlony analysis for the presence of a common oncofoetal protein in organs of rats with DMBA-induced pancreatic cancer. Normal: pancreatic tissue from control rats with only beeswax pellet implant (56 μg); X-ray: small-bowel adenocarcinoma (43 μg); Panc: DMBA-induced pancreatic adenocarcinoma (48 μg) Organs from rats with the DMBA pancreatic cancer Lung (41 μg); Liver (47 μg); Spleen (39 μg) AB: absorbed rabbit antiserum generated gainst the X-ray-induced rat small-bowel cancer at a titre of 1:5.

and DMBA-induced pancreatic (Fig. 3) adenocarcinomas contained the oncofoetal protein in the X-ray-induced small-bowel neoplasm, when the migration studies were run against the antiserum to the X-ray-induced tumour.

Other studies involving DMH and DMBA tumorigenesis have shown these chemicals lack organ specificity. For example, DMH has been shown to induce small-bowel adenocarcinoma (Ward, 1974; Dissin *et al.*, 1975), while the aryl hydrocarbon hydroxylase enzymes necessary for activating the procarcinogen DMBA have been identified in monkey, hamster and rat small intestine (Nebert & Gelboin, 1969). We investigated the possible existence of the oncofoetal protein in other organs of the rats bearing the chemically induced neoplasms. The protein has always been positively identified by the Ouchterlony technique in the liver and lungs of those animals bearing chemically induced colonic (Fig. 4) and pancreatic (Fig. 5) tumours. Many of the kidneys, spleens, and apparently non-involved intestinal tissues also had detectable levels of this protein. About 10–15% of the rats given long-term DMH developed small-bowel adenocarcinomas as well as colonic tumours. Our studies of these small-intestinal neoplasms always indicated the presence of the foetal protein.

Previously, we found the oncofoetal protein in the circulatory system of rats having X-ray-induced small-bowel adenocarcinomas (Stevens *et al.*, 1975, 1976). Its presence in the blood might be one reason for finding the foetal protein in the liver and lungs of animals bearing the DMH-induced colonic and DMBA-induced pancreatic cancers. As in the previous observations, we identified the oncofoetal protein in the serum of rats with either the DMH- or DMBA-induced tumours (Table). In addition, the fluid which was encapsulated by the large DMBA-induced pancreatic lesions was

TABLE.—*Presence of the oncofoetal protein in organs of rats with X-ray or chemically induced tumours*

| | Cancer* | | |
|---|---|---|---|
| Organ | Small bowel (X-ray) | Colon (DMH) | Pancreas (DMBA) |
| Small bowel | + | + | + |
| Colon | − | + | − |
| Pancreas | − | − | + |
| Stomach | − | − | − |
| Liver | − | + | + |
| Lung | − | + | + |
| Spleen | − | − | + |
| Kidney | − | − | + |
| Serum | + | + | + |
| Bile | − | − | + |

[a] Ouchterlony analysis was used to identify the common oncofoetal protein, (+) indicating positive identification in the organs from at least 5 tumour-bearing animals and (−) representing either variable or no detection of the protein. Cellular homogenate concentrations for tissues found to be negative were varied over a range up to 150 μg protein and antiserum titre of 1:5.

found to contain a very high content of this protein. The remaining composition of this fluid has yet to be investigated, but in addition to the foetal protein it contained numerous lymphoid cells. So whether this fluid primarily represents pancreatic juice leaked from the cells owing to the development of the lesion, or simply an irritative response to the presence of the chemical remains to be determined. The control animals, which had only beeswax implanted in the pancreas, never contained such a fluid reservoir, so its formation cannot be attributed to either the surgery or the beeswax. For those tissues which were negative, the cellular homogenate concentrations and antiserum titre were varied to ensure that the appropriate concentrations were being utilized. In addition, antiserum absorptions (10 mg protein/ml) with both the positive and negative tissues were carried out. In all the tests, serum absorptions with tissues reported as positive removed the immunoprecipitin activity, and those reported as negative appeared to have no effect.

A number of studies attempted to determine the tumour specificity of this foetal protein. Again, although our results are qualitative, we only detected this oncofoetal protein in tissues or culture cells obtained from animals with digestive-tissue lesions.

### DISCUSSION

Greenstein, as early as 1945, recognized that a feature of many cancer cells was their foetal characteristic, and stated "tumours tend to converge to common enzymatic patterns that in certain cases resemble those of fetal tissues" (Greenstein 1954). Our present findings suggest that tumours induced in endodermally derived tissue (small bowel, colon and pancreas) produce detectable levels of a common foetal protein. It is important to emphasize that the induction procedures are by apparently different mechanisms, consisting of ionizing radiation, an aldylhydrocarbon (DMH) and a polyaromatic hydrocarbon (DMBA). This would suggest a close relationship at the molecular level between the development of tumours derived from the same embryonic tissue by cellular differentiation. Recently, Uriel has very cleverly examined and discussed the various observations of foetal characteristics in cancer cells, and has noted that one major question is whether such tumours with embryonic properties develop from differentiation of a tissue reserve of stem cells, or by a process of retrodifferentiation (Uriel, 1976). He has noted that the presence of such a reserve of stem cells has not been demonstrated in static or expanding cell populations, whereas in contrast, retrodifferentiation has been revealed in adult hepatocytes. Although our findings do not aid in distinguishing which process may actually occur, they are important in demonstrating that different forms of initiation in different adult tissues that result in cancer may lead to a common phenotypic expression as noted by the detectable levels of the oncofoetal protein.

While it is not yet clear why tumours arising from tissues of common embryonic origin develop similar foetal proteins, one suggested possibility is that they aid the cancer cell in its avoidance of host immune surveillance (Alexander, 1974). Although at present we do not know what role these proteins might play in antitumour immunity, we have established in previous studies of the X-ray-induced neoplasm that there are both blocking factors to antitumour cell-mediated immunity (among which is foetal protein) and specific immunoglobulins (IgG) to the protein (Stevens *et al.*, 1978*a*). It must also be considered that, like α-foetoprotein and human carcinoembryonic antigen, this oncofoetal protein may be a normal cell component which is simply increased in quantity during tumorigenesis. These questions can only be answered when a quantitative assay for oncofoetal protein has been developed. However, in terms of specificity, our results do suggest that the foetal protein exists primarily or exclusively with animals having endodermally derived digestive tumours, as we were unable to identify its presence in rats with spontaneous mammary tumours (ectodermal origin) and asbestos-induced mesotheliomas (mesodermal origin). Nor did nonspecific injury trigger its synthesis, within the failure to detect the protein in the remaining tissue upon a 70% small-bowel resection, even though this procedure produced maximum crypt-cell proliferation (Hanson *et al.*, 1977). Other investigators have shown that the foetal proteins of DMH-induced neoplasms may serve as weak tumour-rejection antigens (Steele & Sjögren, 1977) but whether the presently identified proteins have such a function is unknown.

In summary, the use of antisera generated to X-ray-induced rat small-bowel adenocarcinoma allowed the identification of an apparently unique circulating oncofoetal protein in the radiation-induced neoplasm as well as in chemically induced pancreatic and colonic cancer. This is apparently a general phenomenon, as we have studied over 100 rats with radiation-induced neoplasms, and now 12 with the DMH-induced colonic and 8 with DMBA-induced pancreatic adenocarcinomas. While other studies of carcinoembryonic proteins, such as those carried out on α-foetoprotein (Kroes *et al.*, 1975) have shown an increase on exposure to chemical carcinogens, the present results are the first to demonstrate that apparently the carcinogen type is unimportant, provided the target is digestive tissue. The role of foetal proteins in tumorigenesis is unknown, but studies are being presently initiated to determine what influence the oncofoetal protein identified in these studies may have upon antitumour cell-mediated immunity and to develop quantitative methods of comparisons.

Supported by Grant 1R01-ESCA-0235-9 awarded by National Institute of Environmental Health Sciences, NIH.

### REFERENCES

Alexander, P. (1974) Escape from immune destruction by the host through shedding of surface antigens: Is this a characteristic shared by malignant and embryonic cells? *Cancer Res*, **34,** 2077.

Bandara, S., Reddy, S., Narisawa, T. & 4 others, (1975) Colon carcinogenesis and dimethylhydrazine in germ free rats. *Cancer Res.*, **35,** 287.

Belnap, L. P., Cleveland, P. H., Colmerauer, M. E. M., Barone, R. M. & Pilch, Y. H. (1979) Immunogenecity of chemically induced murine colon cancer. *Cancer Res.*, **39,** 1174.

Coop, K. L., Sharp, J. G., Osborne, J. W. & Zimmerman, G. R. (1974) An animal model for the study of small bowel tumours. *Cancer Res.*, **34,** 1487.

Dissin, J., Mills, L. R., Mains, D. L., Black, O., Jr & Webster, P. D., III (1975) Experimental induction of pancreatic adenocarcinoma in rats. *J. Natl Cancer Inst.*, **55,** 857.

Evans, D. L., Parker, D. V. & Frank, M. K. (1979) Biochemical and physical characterizations of tumor-associated foetal antigens. *Cancer Res.*, **39,** 2006.

Gelder, F. B., Reese, C. J., Moosa, A. R., Hall T. & Hunter, R. (1978) Purification partial characterization and clinical evaluation of a pancreatic oncofetal antigen. *Cancer Res.*, **38,** 313.

Greenstein, J. P. (1945) Enzymes in normal and neoplastic tissues. In *AAAS Research Conference on Cancer*. Ed. Moulton. Washington, DC: AAAS. p. 191.

Hanson, W. R., Osborne, J. W. & Sharp, J. G. (1977) Compensation by the residual intestine resection in rat. II. Influence of post operative time interval. *Gastroenterology*, **72,** 701.

Hirschfeld, J. (1960) Immunoelectrophoresis-procedure and application to the study of group-specific variation in sera. *Sci. Tools*, **7,** 18.

KROES, R., SONTAG, J. M., SELL, S., WILLIAMS, G. M. & WEISBURGER, J. A. (1975) Elevated concentrations of a fetoprotein in rats with chemically induced liver tumors. *Cancer Res.*, **35**, 1214.

MARTIN, F., KNOBEL, S., MARTIN. M. & BORDES, M. (1975) A carcino-foetal antigen located on membrane of cells from rat intestinal carcinoma in culture. *Cancer Res.*, **35**, 33.

NEBERT, D. W. & GELBOIN, H. V. (1969) The *in vivo* and *in vitro* induction of aryl hydrocarbon hydroxylase in mammary cells of different species, tissues, strains and developmental and hormonal states. *Arch. Biochem. Biophys.*, **134**, 67.

OUCHTERLONY, O. (1962) Diffusion-in-gel methods for immunological analysis II. *Prog. Allergy*, **6**, 30.

PRICE, M. R. (1974) Isolation of embryonic antigens from chemically induced rat hepatomas. *Biochem. Soc. Trans.*, **2**, 650.

ROTH, J. A., HOMES, E. C., REISFELD, R. A., SLOCUM, H. K. & MORTON, D. L. (1976) Isolation of a soluble tumor-associated antigen from human melanoma. *Cancer*, **37**, 104.

STEELE, G. JR & SJÖGREN, H. O. (1974) Cross-reacting tumour-associated antigen(s) among chemically induced rat colon carcinomas. *Cancer Res.*, **34**, 1801.

STEELE, G., JR, SJÖGREN, H. O., ROSENGREN, J. E., LINDSTROM, C., LARSSON, A. & LEANODOER, L. (1975*a*) Sequential studies of serum blocking activity in rats bearing chemically induced primary bowel tumours. *J. Natl Cancer Inst.*, **54**, 959.

STEELE, G., JR, SJÖGREN, H. O. & PRICE, M. R. (1975*b*) Tumour-associated and embryonic antigens in soluble fractions of a chemically-induced rat colon carcinoma. *Int. J. Cancer*, **16**, 33.

STEELE, G., JR & SJÖGREN, H. O. (1977) Cell surface antigens in rat colon cancer model: Correlation with inhibition of tumour growth. *Surgery*, **82**, 164.

STEVENS, R. H., ENGLUND, C. W., OSBORNE, J. W., CHENG, H. F. & RICHERSON, A. B. (1975) Oncofetal protein accompanying irradiation-induced small bowel adenocarcinoma in the rat. *J. Natl Cancer Inst.*, **55**, 1011.

STEVENS, R. H., ENGLUND, C. W., OSBORNE, J. W., CHENG, H. F. & HOFFMAN, K. L. (1976) Identification and characterization of a circulating tumor-associated oncofetal protein from a radiation-induced adenocarcinoma of the rat small bowel. *Cancer Res.*, **36**, 3260.

STEVENS, R. H., BROOKS, G. P., OSBORNE, J. W., HOFFMAN, K. L. & LAWSON, A. J. (1978*a*) Lymphocyte cytotoxicity in X-irradiation-induced rat small bowel adenocarcinoma III. Blocking by 3M KCl extract. *J. Immunol.* **120**, 335.

STEVENS, R. H., GRAVES, J. M., MEEK, E. S., OSBORNE, J. W., CHENG, H. F. & LOVEN, D. P. (1978*b*) Cyclic nucleotide concentrations in 7,12-dimethylbenz[a]anthracene induced pancreatic cancer in rats. *J. Natl Cancer Inst.*, **61**, 1281.

STONEHILL, E. H. & BENDICH, A. (1970) Retrogenetic expression: The reappearance of embryonal antigens in cancer cells. *Nature*, **229**, 370.

URIEL, J. (1976) Cancer: Retrodifferentiation and the myth of Faust. *Cancer Res.*, **36**, 4269.

WARD, J. M. (1974) Morphogenesis of chemically induced neoplasms of the colon and small intestine in rats. *Lab. Invest.*, **30**, 505.